# Context specific effects of substrate composition on the taxonomic and functional diversity of macroinvertebrate communities in temperate lowland streams

**DOI:** 10.1002/ece3.70034

**Published:** 2024-08-27

**Authors:** Kate L. Mathers, Patrick D. Armitage, Matthew Hill, Melanie Bickerton, Morwenna Mckenzie, Isabel Pardo, David Tickner, Paul J. Wood

**Affiliations:** ^1^ Geography and Environment Loughborough University Loughborough UK; ^2^ Freshwater Biological Association, River Laboratory Wareham UK; ^3^ Department of Life and Environmental Sciences, Faculty of Science and Technology Bournemouth University Poole UK; ^4^ School of Geography, Earth and Environmental Sciences University of Birmingham Edgbaston UK; ^5^ Department of Ecology and Animal Biology University of Vigo Vigo Spain; ^6^ WWF‐UK, Living Planet Centre Woking UK

**Keywords:** beta diversity, context‐specific, fine sediment, functional traits, mesohabitat, meso‐scale

## Abstract

Substrate composition has been widely recognised as a primary variable shaping lotic macroinvertebrate communities at the habitat unit level. However, fundamental understanding of how communities inhabiting mineralogical habitats (i.e., gravel, sand and silt) are structured across differing rivers is lacking. Moreover, research largely focusses on gravel beds and fine sediment in general (<2 mm) and as a result detailed field observations specifically of the sand and silt fractions are lacking. Using data from five UK streams collated from published studies, we assess taxonomic and functional biodiversity (alpha and beta diversity) at the habitat unit level (as defined by substrate composition of sand, silt and gravel). We found that the composition of taxonomic communities were clearly different in all habitat units for each individual stream (and at the landscape scale), with comparable, but less strong, distinctions between substrates for functional macroinvertebrate community composition. However, alpha diversity metrics and Local Contribution to Beta Diversity (LCBD) recorded among the different habitat units varied significantly across individual rivers, and the amount of variation explained by the habitat unit for taxonomic and functional composition demonstrated considerable differences suggesting strong context dependence. The depositional fine sediment habitats of sand and silt were found to support a discrete community composition and differing levels of alpha and beta diversity within and between rivers. We advocate that care should be taken when seeking to generalise biodiversity patterns at a landscape scale as our study highlights the high degree of context dependency when considering the role of the habitat template. Moreover, our results provide evidence that discriminating between the size fractions of fine sediment habitats (sand or silt) is important to fully elucidate the wider ecological importance of these habitats and the distinct taxonomic and functional biodiversity they support.

## Graphical abstract

We show that river bed substrate composition is instrumental in structuring macroinvertebrate biodiversity but care should be taken when trying to generalise patterns at a landscape scale as context dependence may be present. Sand and silt are often viewed together in fine sediment research (<2 mm) but we show they consistently support different diversity.

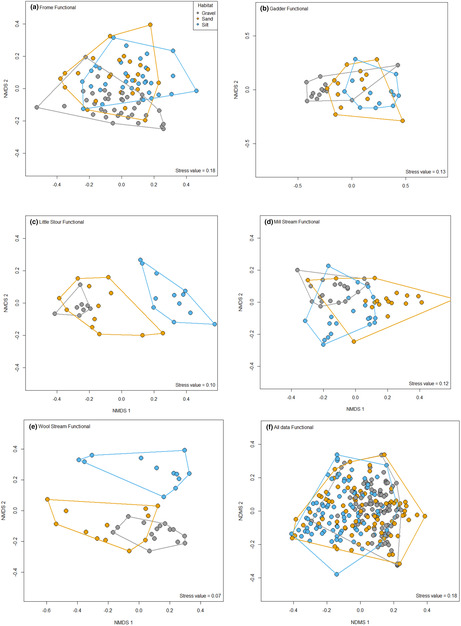

## 1 INTRODUCTION

Rivers and streams are naturally characterised by high levels of spatial and temporal heterogeneity, which in turn supports high levels of biodiversity (Poff & Ward, 1989). Habitat heterogeneity has long been advocated as being of critical importance in the study of ecology (Southwood, 1977) with the concept of habitat patch dynamics historically being fundamental in understanding the distribution of aquatic organisms (Beisel et al., 2000; Pringle et al., 1988; Townsend, 1989). Despite this, much of basic and applied ecological research is still conducted at the meso‐scale (e.g., riffle/pool habitats; Salmaso et al., 2021), including many global biomonitoring programmes that typically aggregate the different mesohabitats sampled (e.g., Environment Agency, 2022; USEPA, 2016; see also review by Buss et al., 2015). Consequently, results from these programmes/studies are unable to inform the mechanistic understanding of how the physical template structures ecological communities.

Recent research has demonstrated that local environmental variability at the habitat scale is a primary driver of richness and compositional variation of lotic aquatic macroinvertebrates (Doretto, Receveur, et al., 2022; Perez Rocha et al., 2018). This local habitat heterogeneity has been recognised to be influential in controlling the outcomes of conservation and restoration activities (Verdonschot et al., 2016; White et al., 2017), flow regulation (Armitage & Pardo, 1995; White et al., 2019), and in assessing habitat suitability for target organisms (Nestler et al., 2019) and riverine health (Kemp et al., 1999; Maddock, 1999). Substrate composition has been acknowledged to be a primary determinant in structuring lotic macroinvertebrate communities (Burdon et al., 2013; Culp et al., 1983; Cummins & Lauff, 1969; Jähnig & Lorenz, 2008) and forms the basis of much research conducted at the habitat scale. Stream ecologists have typically defined distinct habitat units based on their substrate particle size, composition or vegetation cover with a range of terminologies being employed in the literature; mesohabitats (Armitage et al., 1995), functional habitats (Kemp et al., 1999), microhabitats (Frissell et al., 1986) and biotopes (Newson et al., 1998). Despite the various terminologies, all are founded on a similar concept; that habitat units of distinct substrate composition (and their hydraulic characteristics) support discrete macroinvertebrate communities.

Fine sediment (typically referred to as particles <2 mm) is a natural component of riverine ecosystems. However, modifications to rainfall and runoff regimes, in combination with land use changes, have resulted in the delivery of excessive quantities of fine sediment to many lowland river systems (Burt et al., 2016; Collins & Zhang, 2016). Although often delivered to rivers across the entire catchment, fine sediment deposition is often controlled at the local scale via hydraulic conditions (Mathers & Wood, 2016; Schälchli, 1992). Studies examining the role of fine sediment in structuring macroinvertebrate communities at the habitat level, may therefore help identify clearer relationships and be more relevant to the in‐channel management of fine sediment than those currently employing meso‐scale observations (Larsen & Ormerod, 2010), but these studies remain rare (but see Doretto, Espa, et al., 2022; Mathers et al., 2023; Salmaso et al., 2021).

It has been observed that patch‐scale removal of fine sediment in lowland rivers subjected to chronic fine sediment loading resulted in distinct macroinvertebrate communities (Mathers, Rice, & Wood, 2017; Pacioglu et al., 2012). Lowland streams, which are typically naturally characterised by high levels of fine sediment, have also been shown to support high biodiversity at the landscape level, including species of conservation value, where there is distinct habitat provision (Demars et al., 2012; Larsen et al., 2009; Wood & Armitage, 1999). Therefore, understanding the processes affecting biodiversity at the habitat unit level may contribute to development of more clearly focussed conservation and management practices (Heino et al., 2022; Hill et al., 2021).

Much of the research focused on the ecological association of invertebrate communities with fine sediment has employed the definition of all particles constituting <2 mm, without considering the specific size fractions or composition (organic vs inorganic) of fine sediment (but see Blöcher et al., 2020; Demars et al., 2012; Mathers et al., 2023; Von Bertrab et al., 2013). It is highly likely that the different fine sediment fractions of sand (2–0.063 mm as defined by the Wentworth size scale) and silt (<0.063 mm) support different taxonomic and functional communities at the local (habitat) and landscape scale. A recent study by Mathers et al. (2023) demonstrated the importance of discriminating sand and silt fractions particularly given the seasonally variable importance of silt habitats for resource provision (Armitage et al., 1995). However, there remains a deficit of ecological research discriminating fine sediment into sand and silt fractions and whether the two fractions consistently support different macroinvertebrate communities, research required to ensure management practices are effective.

Fine sediment has been reported to act as an environmental filter by selectively removing taxa that are sensitive to fine sediment by impairing trophic resources (Doretto et al., 2017; Rabení et al., 2005), exclusion of larger bodied organisms via infilling of interstitial pore space (Mathers et al., 2019; Peralta‐Maraver et al., 2019), direct clogging of gill apparatus (Mckenzie et al., 2020) and burial of individuals with particular locomotion strategies/habitat preferences (Wood et al., 2005). As such, it is highly likely that different substrate fractions at the habitat level may also support functionally discrete macroinvertebrate communities. Moreover, the ecological implications of fine sediment have recently been shown to be context specific (Mathers et al., 2022; Mckenzie et al., 2024), reflecting past environmental filtering (sensitive species being removed) and river typology/flow regime characteristics. Therefore the biodiversity supported by different mineralogical habitat patches is likely to vary significantly. Context dependency has been defined to occur when a relationship between a variable differs under different spatiotemporal (and ecological) conditions (Catford et al., 2022). Studies examining the relative importance of habitat patches at the level of individual streams are required to understand how the most effective management practices could be employed; whether biodiversity patterns be generalised, or assessed on a river by river basis so that appropriate management can be undertaken (Armitage & Cannan, 2000).

In this study, we assess the relative importance of mineral habitat patches (in this instance gravel, sand and silt) for taxonomic and functional macroinvertebrate biodiversity. By examining taxonomic and functional alpha and beta diversity (including local contribution components) at the habitat level across five lotic systems, we aim to elucidate the mechanistic processes structuring the macroinvertebrate communities of fine sediment habitats at scales relevant for river management and conservation. Our study complements Mathers et al. (2023) by extending our observations to five rivers using collated published data to understand the importance of context dependence. We also aim to add to the relatively sparse existing information pertaining to the importance of fine sediment habitats in supporting biodiversity and its potential wider conservation value as a habitat. Finally we seek to investigate the need to discriminate between fine sediment fractions (silt and sand) in lowland rivers in order to accurately characterise the biodiversity supported. Specifically, we sought to examine the following research questions:

1. Does the taxonomic and functional biodiversity supported by mineralogical habitat patches (gravel, sand and silt) demonstrate strong context dependence across the five studied streams?
2. Is it ecologically important to discriminate between the fine sediment fractions of sand and silt, and is this consistent across all streams?

## 2 MATERIALS AND METHODS

### 2.1 Datasets characterised

Four datasets were collated (originally published in Armitage & Cannan, 2000; Armitage et al., 1995; Pardo & Armitage, 1997; Tickner et al., 2000; Wood et al., 1999) that comprised macroinvertebrate samples collected from distinct substrate habitats in five UK streams; Mill Stream (Dorset), Wool Stream (Dorset), Frome (Dorset), Gadder (Norfolk) and Little Stour (Kent); the two former are tributaries of the River Frome. For further information on individual sites see Appendix 1 and primary source papers; all streams were wadable comprising Strahler stream order 1–3. All studies utilised a similar methodological approach (15‐s kick focused on the habitat patch visually identified prior to sampling or a modified Hess sampler). In each study and stream, discrete substrate patches were visually identified in summer (June–August), and replicate macroinvertebrate samples taken in each habitat unit. Kick samples were conducted on the spot (15‐s) ensuring only the habitat patch visually identified and delineated prior to sampling was sampled, comparable to that of the Hess sampler area. Summer is when the flow regime is most stable in perennial temperate lowland streams resulting in the most pronounced differences between distinct substrate patches, thereby facilitating examination of the biological‐substrate interactions responsible for structuring macroinvertebrate communities without the confounding factor of flow (Mckenzie et al., 2022a; White et al., 2019).

Habitats examined in each study differed but three were common to all studies and retained here: (i) gravel (number of samples = 91), (ii) sand (*n* = 82) and (iii) silt (*n* = 89). These three habitats represent the mineralogical substrate gradient recorded in rivers and were characterised as discrete patches of substrate as opposed to a mixed substrate composition. Due to the different resolutions of identification within the studies, the highest level of taxonomy was employed and all datasets were harmonised to family level, with the exception of Oligochaeta and Hydracarina which were recorded as such. A total of 344 samples were used in the analysis.

### 2.2 Functional traits

Macroinvertebrates were assigned to 11 biological ‘grouping features’ comprising 63 functional traits from the fuzzy coded Tachet et al. (2010) European trait database (Table S1). Trait values were standardised following ‘fuzzy coding’ standardisation (Chevene et al., 1994) using the ‘*prep.fuzzy*’ function in the ade4 R package (Thioulouse et al., 2018), so that each grouping feature summed to 1. As our data were resolved to the family level, affinities of all genera recorded in the trait database were averaged to provide a family score (following Gayraud et al., 2003). A total of 77 taxa were assigned functional traits (of the 80 taxa).

### 2.3 Statistical analyses

#### 2.3.1 Taxonomic and functional alpha diversity associated with habitat classification

Six community level taxonomic and functional metrics were calculated. Three taxonomic metrics comprising community abundance, taxa richness, and Ephemeroptera, Plecoptera and Trichoptera (EPT) richness. Three metrics of functional diversity were also calculated: functional richness (FRic), representing the minimum convex hull encompassing all species, functional evenness (FEve), reflecting the regularity in which species are distributed across functional space and functional divergence (FDiv), representing how abundance is distributed within the volume of functional space occupied by species (Villéger et al., 2008). The three functional diversity metrics were computed using the ‘*dbFD’* function on a Gower dissimilarity matrix in the FD R package (Laliberté et al., 2014).

Statistical differences in the alpha diversity metrics were analysed using a linear model fitted with the fixed interacting factors of river and habitat via the ‘*lm*’ function in the stats package. Where significant differences associated with the interaction of river and habitat occurred post‐hoc pairwise comparisons of habitat groups by individual rivers were performed using estimated marginal means in the emmeans package (Lenth et al., 2024). Differences in the six community metrics for the entire dataset were assessed via linear mixed‐effects models using the ‘*lmer*’ function in lme4 package (Bates et al., 2015) fitted with the fixed effect of habitat and the random effect of stream to reflect spatial autocorrelation. Post‐hoc pairwise comparisons of individual habitat groups were performed as above. All statistical models were validated for normality and homoscedasticity by visually checking residuals (Zuur et al., 2009) and where it was necessary, transformations performed (functional divergence and functional evenness data were log(x + 1) transformed for all models and abundance data log transformed for the linear mixed effect model). All analyses were conducted in the R Environment version 4.1 (R Development Core Team, 2021).

#### 2.3.2 Taxonomic and functional composition associated with habitat classification

Prior to all functional community analyses, the dimensionality of the taxa‐by‐traits matrix was reduced using the Gower distance to provide taxa‐by‐taxa functional distance matrix (‘*gowdis’* function in the FD package), which underwent hierarchical clustering using an unweighted pair group method with arithmetic means (UMPGA in hclust function). Thereafter, following Cardoso et al. (2015), the resulting functional tree was analysed along with the sites‐by‐taxa matrix to provide a functional site‐by‐site dissimilarity matrix, using the *beta* function in the BAT package (Cardoso et al., 2015). Taxonomic (using Bray–Curtis dissimilarity index) and functional (using the above mentioned functional dissimilarity matrix) compositional differences associated with the three substrate habitats were examined via non‐metric multidimensional scaling (NMDS) using the ‘*metaMDS*’ function in the vegan package (Oksanen et al., 2019). To assess the consistency of the patterns recorded this was undertaken on the five individual streams in addition to all data combined. Statistical differences in community composition associated with habitat, stream and their interaction for the entire dataset and by habitat for the individual rivers, were tested via permutational multivariate analysis of variance (PERMANOVA) using the ‘*adonis*’ function in the vegan package. Where significant differences occurred among habitats, pairwise comparisons of differences were performed using the ‘*pairwise.adonis*’ function (Arbizu, 2019). Indicator species for each substrate classification were identified using the ‘*multipatt*’ function within the indicspecies package (De Cáceres et al., 2020). Indicator values of >0.25 were accepted as ecologically relevant and those with a fidelity value of <0.25 removed to exclude rare taxa (De Cáceres et al., 2012; Dufrêne & Legendre, 1997).

#### 2.3.3 Taxonomic and functional beta diversity associated with habitat classification

To test for differences in the heterogeneity of macroinvertebrate community composition (beta diversity) between each (1) habitat, (2) stream and (3) each stream/habitat combination, homogeneity of multivariate dispersions were calculated for functional (using the above mentioned functional dissimilarity matrix) and taxonomic (Bray–Curtis dissimilarity index) communities using the ‘*betadisper*’ function in the vegan R package for all rivers independently and for the entire dataset combined. Statistical differences in multivariate dispersion between habitat groups were tested using one‐way ANOVA. Where significant differences occurred for a habitat, pairwise comparisons of differences were tested via Tukey's post hoc tests. Finally, the local contribution to taxonomic beta diversity (LCBD) was calculated using Hellinger transformed presence‐absence data for each habitat unit across all data using the ‘*betadiv*’ function in the adespatial package (Dray et al., 2022). The LCBD metric quantifies the ecological uniqueness of each sample compared to other samples, with high values indicative of high ecological dissimilarity to other samples (Heino & Grönroos, 2017). Differences in LCBD values between the habitats were tested via Kruskal–Wallis. Where significant differences occurred, pairwise comparisons of differences were tested using Nemenyi post hoc tests using the PMCMRplus package (Pohlert, 2023).

## 3 RESULTS

A total of 101,499 individuals were identified comprising 69 families. Oligochaeta (20%), Chironomidae (19%), Tateidae (14%) and Gammaridae (13%), comprised the most common families recorded. The Frome supported the greatest number of families (58), followed by Mill Stream (53), Wool (44), Gadder (32), and Little Stour (27). Indicator analysis using all data indicated that gravel habitats supported the greatest number of indicator taxa (14), silt supported 11 indicator taxa whilst sand supported only two indicator taxa (Table S2).

### 3.1 Taxonomic and functional alpha diversity associated with habitat classification

**FIGURE 1:**
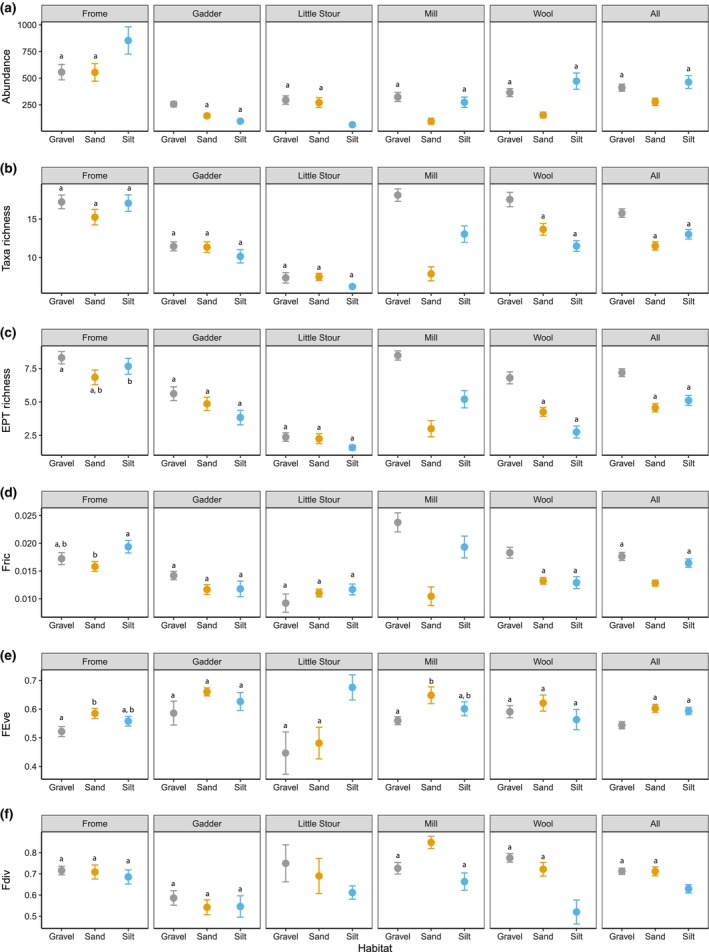
Mean (±1 SE) (a) abundance; (b) taxa richness; (c) EPT richness; (d) functional richness (FRic); (e) functional evenness (FEve); and (f) functional divergence (FDiv) of macroinvertebrate communities in gravel, sand and silt habitats in each of the five streams and for all data combined. Habitats within each river/dataset where no significant differences were recorded are indicated with the same letter.

All alpha diversity metrics demonstrated significant differences among habitats, by stream and by the interaction of stream and habitat (Table S3). Considering all data together, sand habitats demonstrated significantly reduced abundances compared to silt and gravel habitats (Figure 1a; Table S4), and sand and silt habitats supported reduced taxa and EPT richness comparative to gravel habitats (Figure 1b,c; Table S4). Sand habitats supported reduced functional richness relative to both silt and gravel habitats (Figure 1d; Table S4) and greater functional evenness than gravel habitats (Figure 1e; Table S4). Functional divergence was significantly reduced in silt habitats relative to both gravel and sand habitats (Figure 1f; Table S4). However, community metrics demonstrated highly variable patterns dependent on the stream examined with few consistent patterns present.

Community abundance was highly variable among habitats and across rivers (Figure 1; Table S4). Considering richness metrics (taxa, EPT and functional richness), in some instances (Mill Stream), gravel habitats demonstrated the greatest richness values (Figure 1b–d; Table S4), whilst in other instances (Little Stour) there was no difference in richness values between any of the habitats (Figure 1b–d; Table S4). For some streams (Frome, Gadder) there were mixed effects of the habitat on the biodiversity supported, with significant differences exhibited for some richness metrics but no statistical differences for others (Figure 1b–d; Table S4). Communities inhabiting sand (Frome and Mill Stream) demonstrated higher functional evenness than gravel habitats and silt habitats (Little Stour) displayed higher evenness then gravel and sand habitats (Figure 1e; Table S4). In addition, communities in sand habitats in the Mill Stream demonstrated significantly higher functional divergence, while silt habitats in the Wool Stream displayed significantly reduced functional divergence (Figure 1f, Table S4).

### 3.2 Taxonomic and functional composition associated with habitat classification

PERMANOVA analyses of taxonomic and functional community composition indicated that whilst stream identity (Figure S1) explained the greatest amount of variation, habitat also exerted a significant influence (Table 1). For taxonomic communities, habitat explained 9.22% of the variation in composition (relative to 20.04% by river) whilst for functional communities this was 7.24% (relative to 13.87% by river). However, for both diversity facets, the interaction of stream and habitat was significant and explained a moderate amount of variation (10.23% for taxonomic and 9.74% for functional respectively; Table 1).

**TABLE 1:** Summary of PERMANOVA output assessing the relative importance of habitat, stream and their interaction on taxonomic and functional macroinvertebrate communities.

	Taxonomic			Functional		
	*F*	*r* ^2^	*p*	*F*	*r* ^2^	*p*
Habitat type	18.82	9.22	**<.001**	12.93	7.24	**<.001**
Stream	20.46	20.04	**<.001**	12.39	13.87	**<.001**
Habitat type x stream	5.22	10.23	**<.001**	4.35	9.74	**<.001**

*Note*: Significant (*p* < .05) results are in bold.

Considering individual systems, the variation in community composition that habitat explained showed considerable context dependence with the Little Stour and Wool Stream displaying the greatest values (42.96% and 34.98% for taxonomic and 52.76% and 30.94% for functional respectively; Table 2). Nevertheless, all streams demonstrated significant differences in community composition associated with habitat (all accounting for >14% of variation; Table 2). In all streams, taxonomic community composition was more strongly associated with the habitat template relative to functional community composition (Table 2).

**TABLE 2:** Summary of PERMANOVA output assessing the relative importance of habitat on taxonomic and functional macroinvertebrate communities for each individual stream.

Stream	Taxonomic			Functional		
	*F*	*r* ^2^	*p*	*F*	*r* ^2^	*p*
Frome	7.66	14.41	**<.001**	4.03	8.13	**<.001**
Gadder	8.12	31.09	**<.001**	6.74	27.26	**<.001**
Little Stour	10.92	42.96	**<.001**	7.80	34.98	**<.001**
Mill Stream	7.88	22.23	**<.001**	6.52	19.16	**<.001**
Wool Stream	10.47	36.78	**<.001**	8.07	30.94	**<.001**

*Note*: Significant (*p* < .05) results are in bold.

**FIGURE 2:**
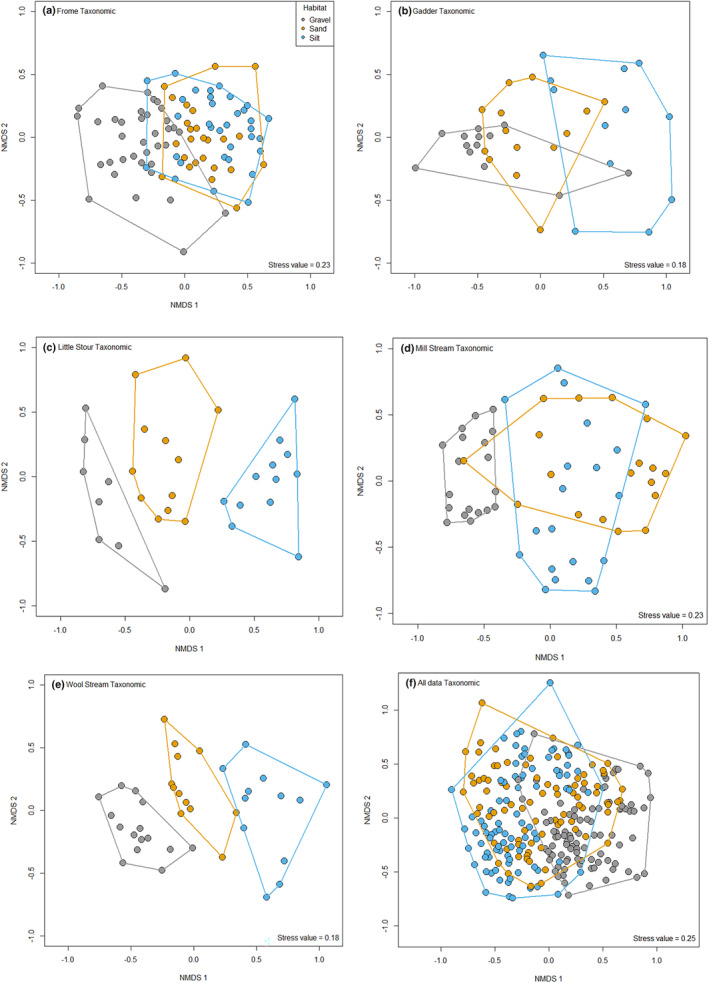
Non‐metric multidimensional scaling (NMDS) of taxonomic macroinvertebrate community composition associated with gravel, sand and silt habitats from (a) Frome; (b) Gadder; (c) Little Stour; (d) Mill Stream; (e) Wool Stream; and (f) all data combined.

**FIGURE 3:**
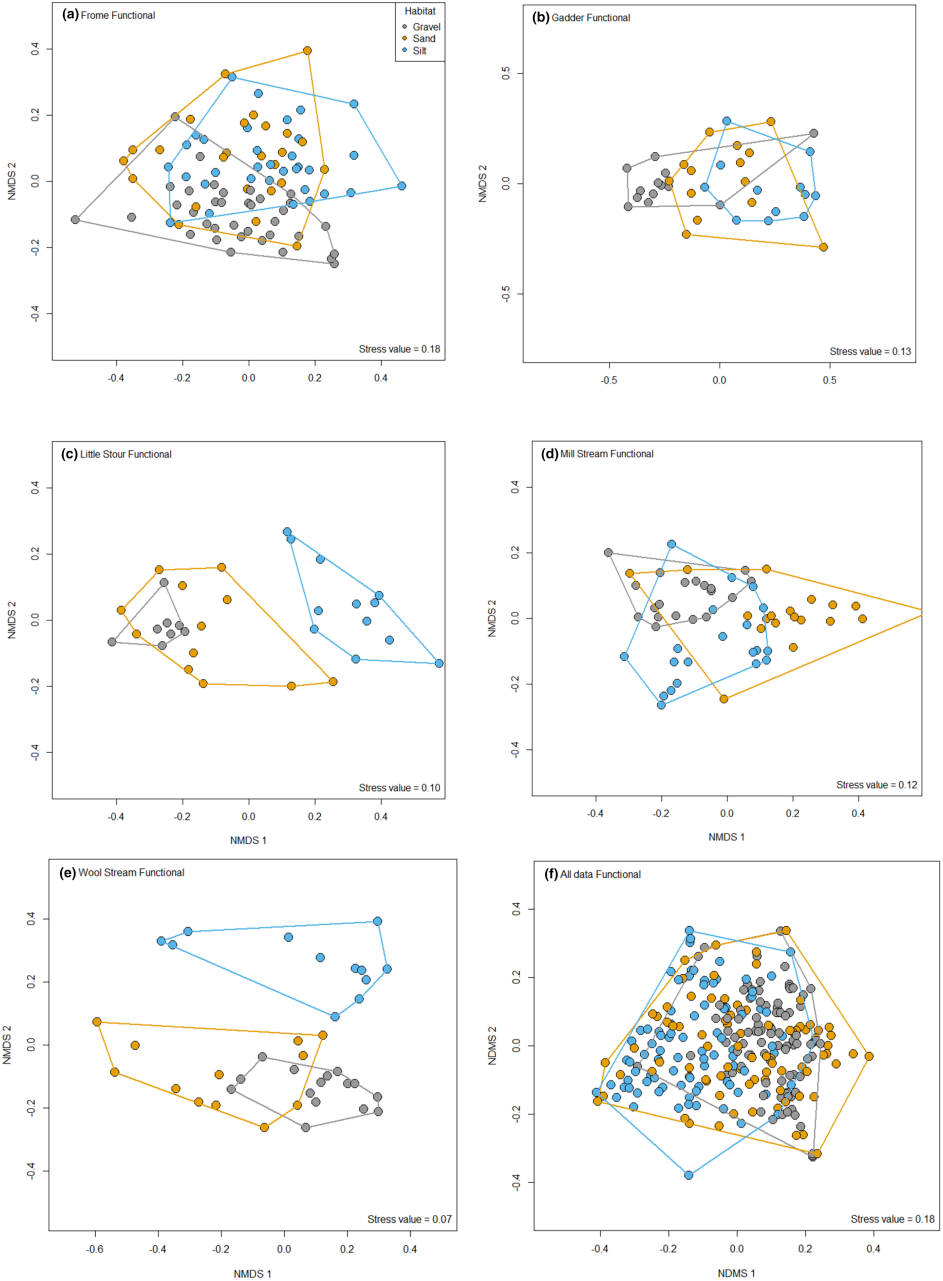
Non‐metric multidimensional scaling (NMDS) of functional macroinvertebrate community composition associated with gravel, sand and silt habitats from (a) Frome; (b) Gadder; (c) Little Stour; (d) Mill Stream; (e) Wool Stream; and (f) all data combined.

When pairwise differences in community composition for all data together were considered, visual ordinations indicated that the differences in taxonomic and functional composition for sand and silt habitats were less evident, although PERMANOVA indicated all pairwise differences were significant (Table 3, Figures 1f and 2f). However, pairwise *r* ^2^ values, highlighted that silt and gravel habitats explained the greatest amount of variation (11.75% for taxonomic and 14.44% functional respectively) with the other two habitat pairwise comparisons accounting for ~5% of variation for taxonomic and 2%–10% for functional communities. There were, however, notable differences between streams in terms of the composition of macroinvertebrates supported. Taxonomic communities demonstrated much clearer and statistically stronger differences in composition, with communities in all three habitats (silt, sand and gravel) being different to each other for all streams (Figure 2; Table 3), except for communities inhabiting sand and silt in the Frome (Figure 2a; Table 3). In contrast, although significant differences were evident in functional community composition, these were less marked and less significant (Figure 3; Table 3). The Gadder, Mill Stream and Wool Stream demonstrated significant differences in functional community composition between all three habitats, with the functional communities sand and silt being similar in the Frome (Figure 3; Table 3).

**TABLE 3:** Summary of pairwise PERMANOVA testing for statistical differences between habitat types for each individual stream.

Taxonomic	Sand	Silt	Functional	Sand	Silt
*Frome*			*Frome*		
Gravel	**0.001**	**0.001**	Gravel	**0.003**	**0.003**
Sand		0.304	Sand		0.649
*Gadder*			*Gadder*		
Gravel	**0.001**	**0.001**	Gravel	**0.014**	**0.001**
Sand		**0.002**	Sand		**0.003**
*Little Stour*			*Little Stour*		
Gravel	**0.018**	**0.001**	Gravel	**0.012**	**0.001**
Sand		**0.001**	Sand		**0.001**
*Mill Stream*			*Mill Stream*		
Gravel	**0.001**	**0.001**	Gravel	**0.001**	**0.003**
Sand		**0.001**	Sand		**0.001**
*Wool Stream*			*Wool Stream*		
Gravel	**0.001**	**0.001**	Gravel	**0.002**	**0.001**
Sand		**0.001**	Sand		**0.004**
*All data*			*All data*		
Gravel	**0.001**	**0.001**	Gravel	**0.003**	**0.003**
Sand		**0.001**	Sand		**0.003**

*Note*: Significant (*p* < .05) results are in bold.

### 3.3 Taxonomic and functional beta diversity associated with habitat classification

Significant differences were evident among the habitats (taxonomic *F*_2,259_ = 11.60, *p* ≤ .001; functional *F*_2,259_ = 24.74, *p* ≤ .001) when total beta diversity for all systems were considered, for taxonomic and functional communities. Overall, communities in silt (*p* = .001 for taxonomic and *p* ≤ .001 for functional) and sand (*p* < .001 for both diversity facets) habitats demonstrated significantly greater heterogeneity than those inhabiting gravel habitats (Figure S2a,d). Communities inhabiting sand demonstrated the greatest heterogeneity (mean taxonomic multivariate dispersion distance: 0.557 functional: 0.569) followed by silt (taxonomic: 0.538 and functional: 0.568) and lastly gravel (taxonomic: 0.505, and functional: 0.508) regardless of the biodiversity facet examined. However, there were stream specific differences in taxonomic and functional community heterogeneity associated with all three habitats (Table S5 and Figure S3) and by stream identity for both taxonomic (*F*_4,257_ = 24.36, *p* ≤ .001) and functional (*F*_4,257_ = 11.86, *p* ≤ .001) community composition (Table S6 and Figure S2b,d).

The Little Stour's taxonomic communities (*F*_2,29_ = 6.133, *p* = .006) and functional communities (*F*_2,29_ = 5.39, *p* = .010) demonstrated significant differences in heterogeneity associated with the three habitats. Post‐hoc comparisons indicated that taxonomic communities inhabiting sand (*p* = .004 taxonomic, *p* = .010 functional) and silt (*p* = .05 taxonomic, *p* = .033 functional) were more heterogenous than those in gravel in the Little Stour and functional communities inhabiting silt (*p* = .007) were much more heterogeneous than those in gravel for the Mill Stream.

**FIGURE 4:**
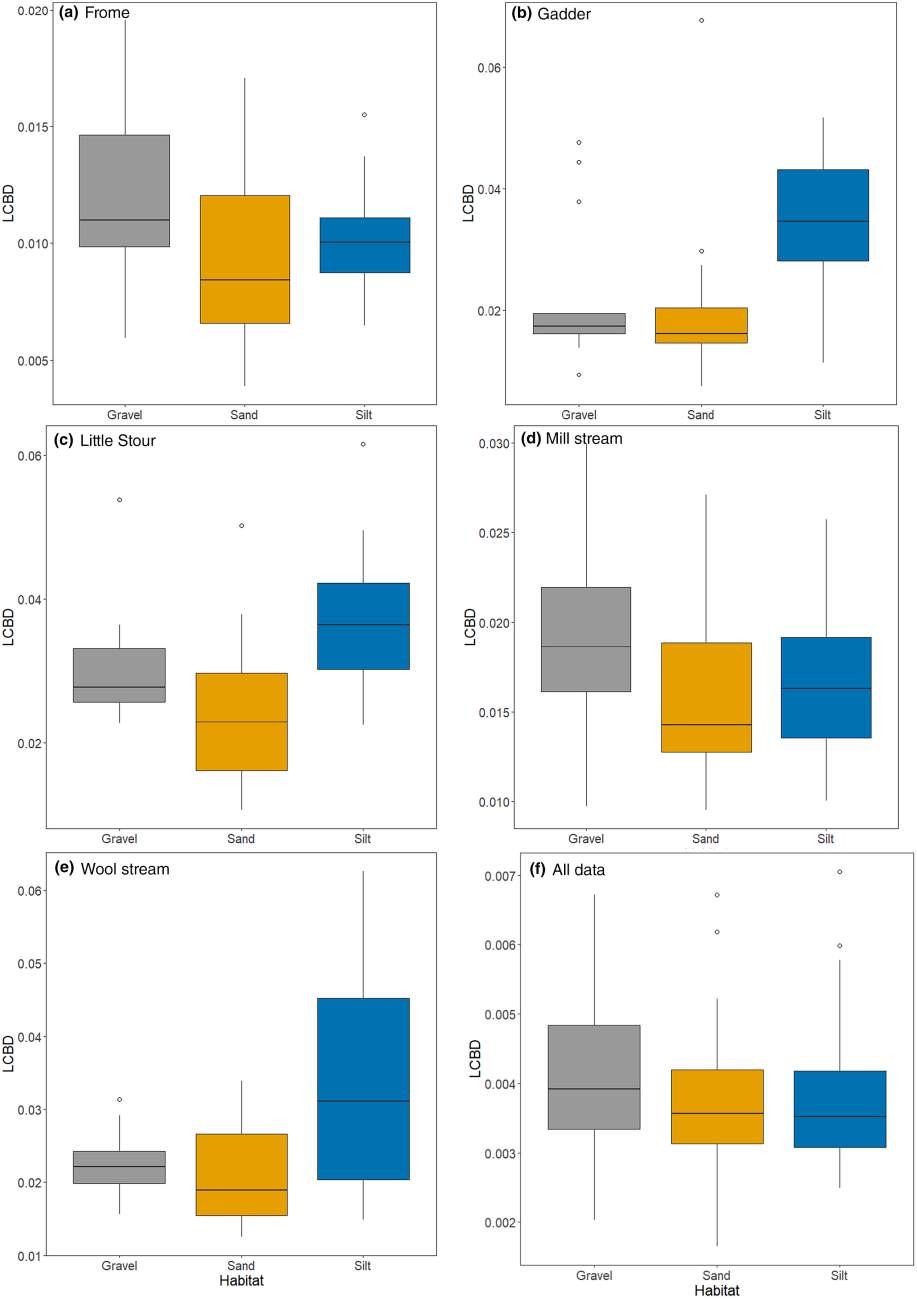
Median local contribution to beta diversity values recorded from each habitat type for (a) Frome; (b) Gadder; (c) Little Stour; (d) Mill Stream; (e) Wool Stream; and (f) all data. Boxes show the 25th, 50th and 75th percentiles, whiskers the 5th and 95th percentiles.

Across the entire dataset, 12 samples recorded significant LCBD values (i.e., contributed significantly to total beta diversity) with seven from gravel habitats, three from silt and two from sand. There was a significant difference in LCBD values among the three habitats when all data were considered (*χ* ^2^_2,559_ = 8.89, *p* = .012; Table S7), with gravel supporting significantly greater LCBD values than both sand and silt habitats (Table S8; Figure 4f). When individual streams were considered, significant differences in LCBD values by habitat were evident for four streams; Frome, Gadder, Little Stour and Wool Stream (*p* < .05; Table S7; Figure 4). For the Gadder, Little Stour and Wool Stream, silt habitats supported the greatest LCBD values (which were statistically greater than sand habitats for all three rivers, and additionally for gravel habitats in Wool Stream; Table S8), whilst for the Frome, gravel habitats supported the highest LCBD values (Figure 4). Six significant LCBD values (*p* < .05) were recorded in the Frome (five gravel, one sand), two for Gadder (one sand, one silt), four for the Mill Stream (two sand, two gravel) and three for the Wool Stream (three silt) when the rivers were considered separately.

## 4 DISCUSSION

Our results indicate that substrate composition, here defined at the habitat unit level, is highly influential in determining the taxonomic and functional composition of macroinvertebrate communities in lowland streams. For both biodiversity facets, the individual stream explained the greatest amount of variation in community composition, however habitat type and the interaction of both factors were significant. Substrate composition at the patch (Beisel et al., 2000; Larsen et al., 2009, 2011) and riffle scale (Davy‐Bowker et al., 2006; Mathers, Hill, & Wood, 2017) has been acknowledged to play an influential role in structuring community composition. However, we found that the role of substrate composition in determining macroinvertebrate biodiversity, was highly variable across the rivers studied, in addition to the biodiversity facet examined (taxonomic or functional).

Silt and sand substrate patches supported different taxonomic and functional composition in all but two (of 12) instance(s). These structural differences in communities inhabiting sand and silt habitat units (regardless of the biodiversity facet), provides further evidence for the call by Mathers et al. (2023) that fine sediment should not be generalised as <2 mm but should be discriminated into its individual fractions of sand and silt (achievable by visual classification) to more accurately represent the communities and ecological processes supported. Blöcher et al. (2020) recorded experimentally that the sediment grain size mattered for certain species although did not examine differences between organic and inorganic matter, whilst Von Bertrab et al. (2013) concluded that the composition of fine sediment (organic content) mattered more than the mass of fine sediment. Indeed, studies incorporating the proportion of organic matter have reported that some components of the ecological community react more strongly and that this explained more of the variation in the community structure (Murphy et al., 2015). Despite the strong evidence that the size classification of fine sediment matters, studies continue to classify fine sediment under the general ‘label’ of particles <2 mm. Given that many biomonitoring programmes globally already visually quantify data using the categories of clay, sand and silt (e.g., Environment Agency, 2022; USEPA, 2016), it would make ecological sense to identify the implications of these fractions independently, rather than pooling the data together.

Beta diversity demonstrated low variation at the individual river level with only one (of five) stream displaying a significant difference in beta diversity values for taxonomic and functional communities (which was also the same river in both instances – Little Stour). In both cases, fine sediment habitats (sand and silt), demonstrated greater heterogeneity than gravel habitats. However, at the landscape scale (when all streams were considered together), taxonomic and functional beta diversity values showed significant variation, predominantly driven by high heterogeneity in sand habitats followed closely by silt. Fine sediment depositional habitats are likely to be spatially and temporally variable as a result of their strong association with the flow regime and near‐bed hydraulics (Wilkes et al., 2017). During low base‐flow conditions, as examined in this study, fine sediment will be readily deposited creating fine sediment zones which will be subsequently scoured once flow increases (Wood & Petts, 1999). Fine sediment zones are therefore highly variable in terms of their substrate composition (sediment depth, % of detritus), and longevity, which potentially leads to highly heterogenous communities relative to other habitats. In marked contrast, communities inhabiting gravel demonstrated a relatively homogenous community at the river and landscape scale.

We observed that the fine sediment depositional habitats composed of silt made an important contribution to LCBD in three of the five rivers, suggesting that silt habitats may support highly unique macroinvertebrate compositions. Silt habitats supported considerably greater numbers of indicator families (11 taxa) than sand habitats (2 taxa). Sand habitats in contrast demonstrated a low contribution to LCBD in all studied rivers, providing further evidence that discriminating between the size categories of fine sediment is vital to elucidate the ecological role of fine sediment in lotic systems. Differences in LCBD values among habitats were however recorded between the study systems, especially for silt habitats, which indicates that the role of fine sediment in lotic systems may be context specific, and management strategies should reflect this (Mathers et al., 2022; Roper et al., 2002). However, we observed that gravel habitats supported the greatest mean LCBD values at the landscape scale, supporting the supposition that gravel is often perceived to be optimal relative to fine sediment habitats.

When alpha diversity was examined, we observed a high degree of context dependence, with high variation in taxonomic and functional alpha diversity across the study streams. Fine sediment is widely reported to selectively filter out sensitive taxa (Jones et al., 2012; Wood & Armitage, 1997) and therefore greater richness could be expected in gravel habitats. However, recent research has begun to demonstrate the context dependent nature of fine sediment deposits for macroinvertebrate communities with Mathers et al. (2022) recording no difference in richness metrics (taxa, EPT and functional richness) in two lowland rivers when macroinvertebrate communities were exposed to clean or heavily sedimented gravels. Subsequently Mathers et al. (2023) demonstrated strong differences in richness metrics in spring for silt deposits relative to gravel habitats, but as occupancy of silt deposits increased over the year, no differences were evident relative to gravel in some richness metrics (EPT, taxa, functional) during summer and autumn.

Alpha diversity results recorded provide further evidence for our hypothesis that sand and silt habitats support differing macroinvertebrate communities and should be discriminated as such, with a number of significant differences in alpha diversity metrics evident between the two depositional habitats. At the individual and landscape level, the two fine sediment habitats typically supported different levels of biodiversity. At the landscape level, sand communities were most impoverished, followed by silt habitats and gravel habitats supported the richest taxonomic communities. Interestingly, no differences were observed for functional or EPT richness between silt and gravel habitats at the landscape scale, suggesting that silt habitats may not be as functionally impoverished as previously assumed.

At the functional level, reduced functional divergence was recorded in silt habitats relative to other habitats at the landscape scale. High functional divergence has been suggested to represent a high degree of niche differentiation with resource efficiency being high (Mason et al., 2005). It is therefore likely that silt habitats are characterised by enhanced levels of resource competition due to the low habitat complexity. In contrast, functional evenness at the landscape scale was found to be higher in sand than gravel habitats suggesting that although the trait niche is occupied in sand, certain parts are underutilised which reflects the relatively impoverished communities recorded. Our results support those of Juvigny‐Khenafou et al. (2021) who observed that functional evenness increased when fine sediment was added to an ex‐situ experiment, whilst functional divergence declined. However, it should be noted that as with all alpha diversity metrics, context dependence between rivers was highly evident in our study. This context dependence likely reflects the regional species pool alongside local abiotic and spatial factors.

### 4.1 Wider implications

Our results highlight the importance of context dependence in the structuring of macroinvertebrate communities by the physical environment. Historically, there has been an emphasis on generalising the role of the environmental template and associated environmental stressors (e.g., flow alteration, pollution) or modifications (e.g., restoration) for ecological communities. However, a growing body of research has advocated the importance of context dependence (Heino et al., 2012; Tonkin et al., 2016), with several papers specifically addressing this issue in recent years (Mathers et al., 2022; Nguyen et al., 2023; Palt et al., 2023; Snåre et al., 2024). By examining one of the fundamental physical habitat characteristics that structures macroinvertebrate communities, we have shown that alpha diversity and LCBD supported by the different habitat units (gravel, sand and silt) varies significantly across individual rivers. In general, taxonomic communities supported discrete communities regardless of the habitat in each individual river, with comparable, but less strong, distinctions between habitats for functional macroinvertebrate communities. We advocate that care should be taken when looking to generalise biodiversity patterns at a landscape scale as there may be differing patterns present in individual rivers which contrasts with landscape scale patterns (for example averaging out patterns when data are combined).

We also found further compelling evidence (following Mathers et al., 2023) to support the need to discriminate fine sediment deposits into the sand or silt fractions, to fully elucidate the ecological implications of fine sediment for macroinvertebrate communities. Further research is required to identify the mechanisms responsible for the observed differences as it may associated with the particle size of the sediment (Blöcher et al., 2020; Mathers et al., 2019), differences in the proportion the organic and inorganic components and therefore trophic resources (Mckenzie et al., 2022b; Murphy et al., 2015) or more likely a combination of the two processes interacting. Regardless of the mechanisms, it's clear that generalising fine sediment as <2 mm is an oversimplification and may mask some of the underlying ecological processes structuring lotic macroinvertebrate communities.

## AUTHOR CONTRIBUTIONS

**Kate L. Mathers:** Conceptualization (lead); data curation (lead); formal analysis (lead); funding acquisition (lead); methodology (equal); project administration (lead); visualization (equal); writing – original draft (lead); writing – review and editing (equal). **Patrick D. Armitage:** Conceptualization (supporting); investigation (equal); methodology (equal); writing – review and editing (equal). **Matthew Hill:** Conceptualization (supporting); formal analysis (supporting); visualization (equal); writing – review and editing (equal). **Melanie Bickerton:** Investigation (equal); writing – review and editing (supporting). **Morwenna Mckenzie:** Writing – review and editing (supporting). **Isabel Pardo:** Investigation (equal); writing – review and editing (supporting). **David Tickner:** Investigation (equal); writing – review and editing (supporting). **Paul J. Wood:** Conceptualization (equal); investigation (equal); methodology (equal); writing – review and editing (equal).

## CONFLICT OF INTEREST STATEMENT

The authors declare no conflict of interest.

## Supporting information


**Data S1.** Supporting information.

## Data Availability

Data (Mathers et al., 2024) are available from Loughborough University Research Repository: DOI: 10.17028/rd.lboro.26397802.
