# Supplementary material for: Context specific effects of substrate composition on the taxonomic and functional diversity of macroinvertebrate communities in temperate lowland streams
Source: Ecol Evol. 2024 Aug 27;14(8):e70034. doi: 10.1002/ece3.70034 (PMC11349607; doi:10.1002/ece3.70034)
Supplement: Supplementary file 1 — Data S1. Supporting information. [file ECE3-14-e70034-s001.zip › Appendix 1_site_details_revised1.docx]

**Appendix 1:** Sample collection and processing methods of the individual datasets

**Armitage et al., 1995, Pardo and Armitage, 1997**

Samples were collected from the Mill Stream in Dorset over a 300 m reach via a 15-second kick / sweep sample using a 900 µm pond net in 1992. 10 replicates were taken for each mesohabitat. Mesohabitats examined in this study were sand, silt, and gravel ‘fast’ (taken as gravel). In total 30 samples taken during the summer were included in this study. Samples were identified to species level where possible.

**Wood et al., 1999**

Samples were collected from three streams; River Gadder (Norfolk), the Little Stour (Kent) and the Mill Stream (Dorset) in 1994. Samples were collected over a 150 m reach via cylinder sampler (0.05m^2^) fitted with a 250 µm net over a 1-minute period. Mesohabitats examined in this study were sand, silt, mixed gravel / sand and gravel. The number of replicates varied dependent on the mesohabitat proportions present at the time. A total of 99 samples were included in this study (Gadder = 39, Mill Stream = 28, Little Stour = 32). Samples were identified to species level where possible, with the exception of Chironomidae and Oligochaeta recorded as such and Baetidae resolved to genus level.

**Tickner et al., 2000**

Samples were collected from the Wool Stream in Dorset over five reaches (of variable lengths) that constituted a 1.25 km stretch. Sampling was conducted via a 15-second kick / sweep sample using a 900 µm pond net in 1997. Samples were identified to family level.

**Armitage and Cannon, 2000**

Samples were collected from the River Frome in Dorset over four 100 m reaches. Sampling was conducted via a 15-second kick / sweep sample using a 900 µm pond net and was repeated over three years; 1995, 1996, 1997. Mesohabitats examined in this study were sand, silt, emergent vegetation (taken as vegetation) and main channel gravel (taken as gravel). A total of 94 samples were included in this study. Samples were identified to family level.

**References**

Armitage, P.D. and Cannan, C.E., 2000. Annual changes in summer patterns of mesohabitat distribution and associated macroinvertebrate assemblages. *Hydrological Processes*, *14*, 3161-3179.

Armitage, P.D., Pardo, I. and Brown, A., 1995. Temporal constancy of faunal assemblages in 'mesohabitats'-application to management?. *Archiv für Hydrobiologie*, 367-387.

Pardo, I. and Armitage, P.D., 1997. Species assemblages as descriptors of mesohabitats. *Hydrobiologia*, *344*, 111-128.

Tickner, D., Armitage, P.D., Bickerton, M.A. and Hall, K.A., 2000. Assessing stream quality using information on mesohabitat distribution and character. *Aquatic Conservation: Marine and Freshwater Ecosystems*, *10*, 179-196.

Wood, P.J., Armitage, P.D., Cannan, C.E. and Petts, G.E., 1999. Instream mesohabitat biodiversity in three groundwater streams under base-flow conditions. *Aquatic Conservation: Marine and Freshwater Ecosystems*, *9*, 265-278.
