# Supplementary material for: Context specific effects of substrate composition on the taxonomic and functional diversity of macroinvertebrate communities in temperate lowland streams
Source: Ecol Evol. 2024 Aug 27;14(8):e70034. doi: 10.1002/ece3.70034 (PMC11349607; doi:10.1002/ece3.70034)
Supplement: Supplementary file 1 — Data S1. Supporting information. [file ECE3-14-e70034-s001.zip › Mesohabitat_Apr_2024_supplementary_revised_traits_jani.pptx]

## Slide 1
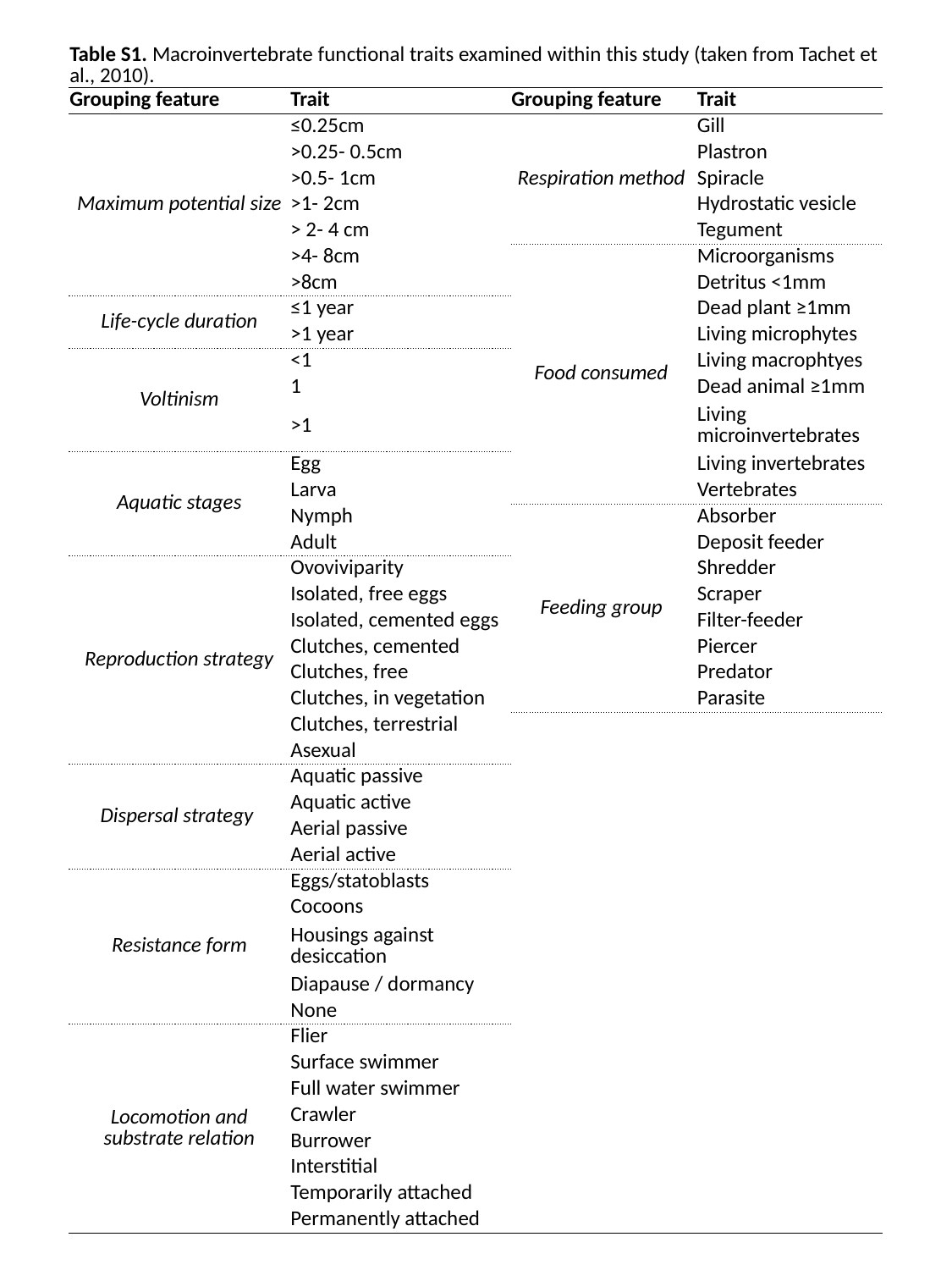

| Table S1. Macroinvertebrate functional traits examined within this study (taken from Tachet et al., 2010). | | | |
| --- | --- | --- | --- |
| Grouping feature | Trait | Grouping feature | Trait |
| Maximum potential size | ≤0.25cm | Respiration method | Gill |
| | >0.25- 0.5cm | | Plastron |
| | >0.5- 1cm | | Spiracle |
| | >1- 2cm | | Hydrostatic vesicle |
| | > 2- 4 cm | | Tegument |
| | >4- 8cm | Food consumed | Microorganisms |
| | >8cm | | Detritus <1mm |
| Life-cycle duration | ≤1 year | | Dead plant ≥1mm |
| | >1 year | | Living microphytes |
| Voltinism | <1 | | Living macrophtyes |
| | 1 | | Dead animal ≥1mm |
| | >1 | | Living microinvertebrates |
| Aquatic stages | Egg | | Living invertebrates |
| | Larva | | Vertebrates |
| | Nymph | Feeding group | Absorber |
| | Adult | | Deposit feeder |
| Reproduction strategy | Ovoviviparity | | Shredder |
| | Isolated, free eggs | | Scraper |
| | Isolated, cemented eggs | | Filter-feeder |
| | Clutches, cemented | | Piercer |
| | Clutches, free | | Predator |
| | Clutches, in vegetation | | Parasite |
| | Clutches, terrestrial | | |
| | Asexual | | |
| Dispersal strategy | Aquatic passive | | |
| | Aquatic active | | |
| | Aerial passive | | |
| | Aerial active | | |
| Resistance form | Eggs/statoblasts | | |
| | Cocoons | | |
| | Housings against desiccation | | |
| | Diapause / dormancy | | |
| | None | | |
| Locomotion and substrate relation | Flier | | |
| | Surface swimmer | | |
| | Full water swimmer | | |
| | Crawler | | |
| | Burrower | | |
| | Interstitial | | |
| | Temporarily attached | | |
| | Permanently attached | | |

## Slide 2
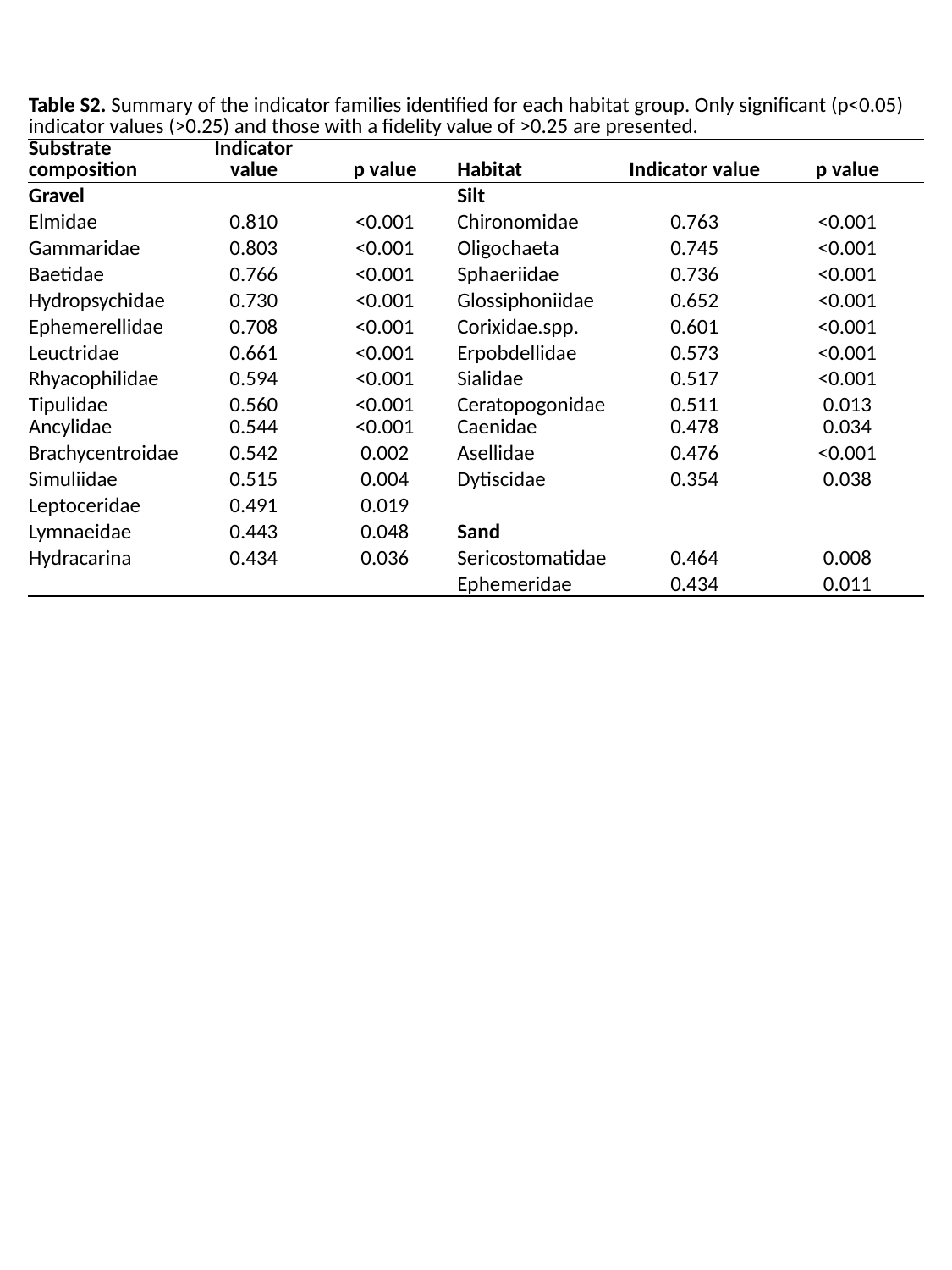

| Table S2. Summary of the indicator families identified for each habitat group. Only significant (p<0.05) indicator values (>0.25) and those with a fidelity value of >0.25 are presented. | | | | | |
| --- | --- | --- | --- | --- | --- |
| Substrate composition | Indicator value | p value | Habitat | Indicator value | p value |
| Gravel | | | Silt | | |
| Elmidae | 0.810 | <0.001 | Chironomidae | 0.763 | <0.001 |
| Gammaridae | 0.803 | <0.001 | Oligochaeta | 0.745 | <0.001 |
| Baetidae | 0.766 | <0.001 | Sphaeriidae | 0.736 | <0.001 |
| Hydropsychidae | 0.730 | <0.001 | Glossiphoniidae | 0.652 | <0.001 |
| Ephemerellidae | 0.708 | <0.001 | Corixidae.spp. | 0.601 | <0.001 |
| Leuctridae | 0.661 | <0.001 | Erpobdellidae | 0.573 | <0.001 |
| Rhyacophilidae | 0.594 | <0.001 | Sialidae | 0.517 | <0.001 |
| Tipulidae | 0.560 | <0.001 | Ceratopogonidae | 0.511 | 0.013 |
| Ancylidae | 0.544 | <0.001 | Caenidae | 0.478 | 0.034 |
| Brachycentroidae | 0.542 | 0.002 | Asellidae | 0.476 | <0.001 |
| Simuliidae | 0.515 | 0.004 | Dytiscidae | 0.354 | 0.038 |
| Leptoceridae | 0.491 | 0.019 | | | |
| Lymnaeidae | 0.443 | 0.048 | Sand | | |
| Hydracarina | 0.434 | 0.036 | Sericostomatidae | 0.464 | 0.008 |
| | | | Ephemeridae | 0.434 | 0.011 |

## Slide 3
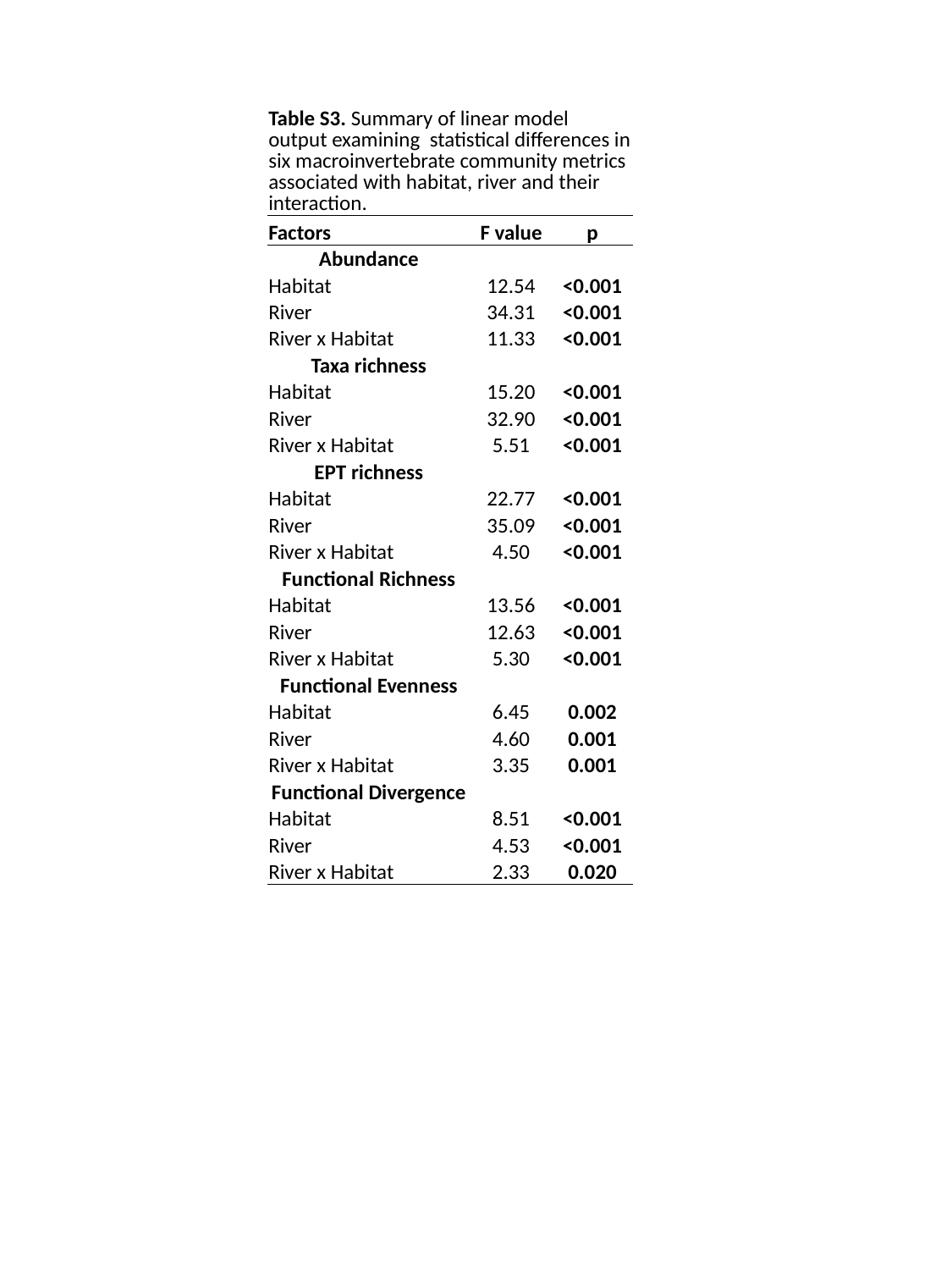

| Table S3. Summary of linear model output examining statistical differences in six macroinvertebrate community metrics associated with habitat, river and their interaction. | | |
| --- | --- | --- |
| Factors | F value | p |
| Abundance | | |
| Habitat | 12.54 | <0.001 |
| River | 34.31 | <0.001 |
| River x Habitat | 11.33 | <0.001 |
| Taxa richness | | |
| Habitat | 15.20 | <0.001 |
| River | 32.90 | <0.001 |
| River x Habitat | 5.51 | <0.001 |
| EPT richness | | |
| Habitat | 22.77 | <0.001 |
| River | 35.09 | <0.001 |
| River x Habitat | 4.50 | <0.001 |
| Functional Richness | | |
| Habitat | 13.56 | <0.001 |
| River | 12.63 | <0.001 |
| River x Habitat | 5.30 | <0.001 |
| Functional Evenness | | |
| Habitat | 6.45 | 0.002 |
| River | 4.60 | 0.001 |
| River x Habitat | 3.35 | 0.001 |
| Functional Divergence | | |
| Habitat | 8.51 | <0.001 |
| River | 4.53 | <0.001 |
| River x Habitat | 2.33 | 0.020 |

## Slide 4
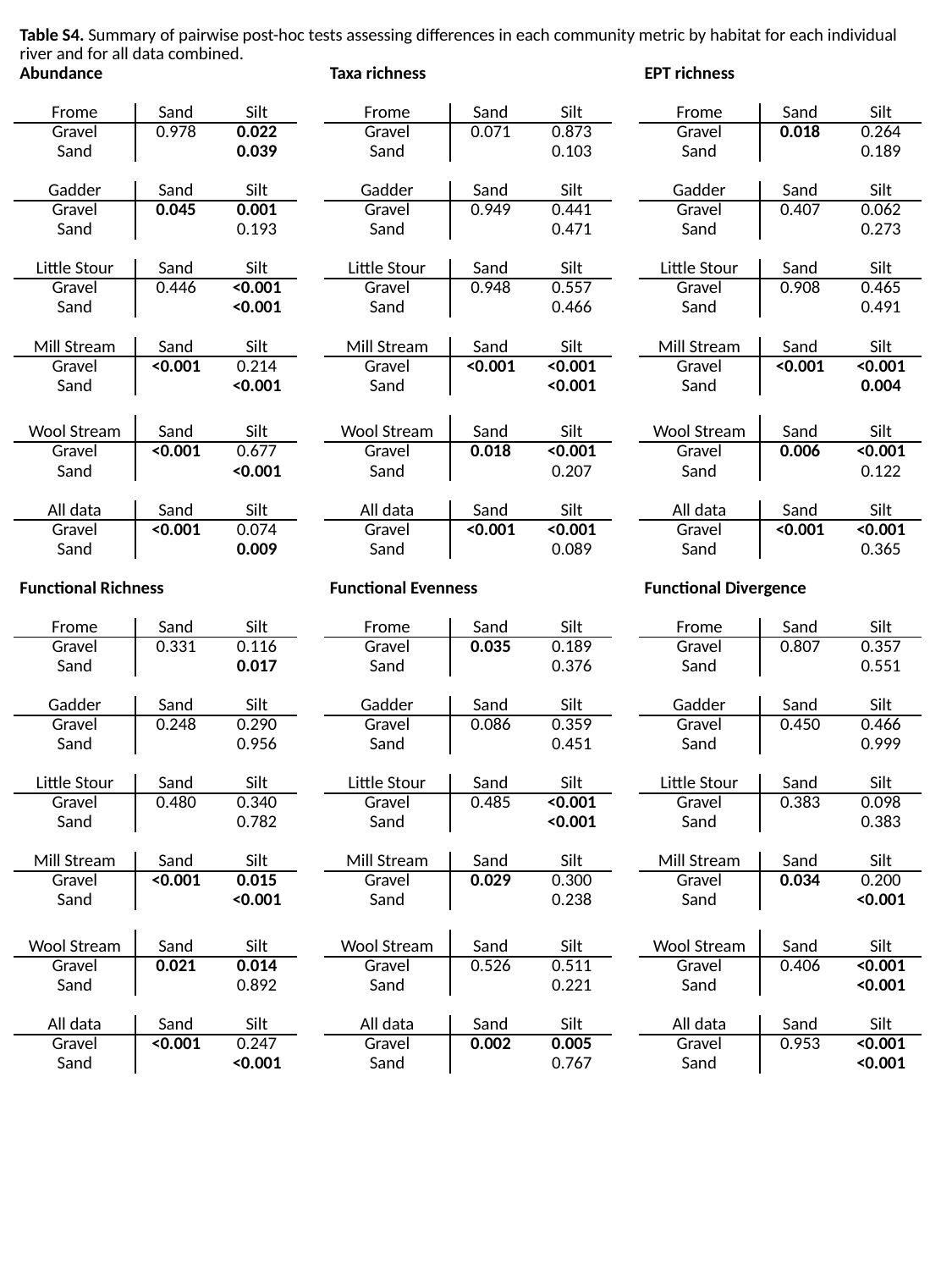

| Table S4. Summary of pairwise post-hoc tests assessing differences in each community metric by habitat for each individual river and for all data combined. | | | | | | | | | | | |
| --- | --- | --- | --- | --- | --- | --- | --- | --- | --- | --- | --- |
| Abundance | | | | Taxa richness | | | | EPT richness | | | |
| | | | | | | | | | | | |
| Frome | Sand | Silt | | Frome | Sand | Silt | | Frome | Sand | Silt | |
| Gravel | 0.978 | 0.022 | | Gravel | 0.071 | 0.873 | | Gravel | 0.018 | 0.264 | |
| Sand | | 0.039 | | Sand | | 0.103 | | Sand | | 0.189 | |
| | | | | | | | | | | | |
| Gadder | Sand | Silt | | Gadder | Sand | Silt | | Gadder | Sand | Silt | |
| Gravel | 0.045 | 0.001 | | Gravel | 0.949 | 0.441 | | Gravel | 0.407 | 0.062 | |
| Sand | | 0.193 | | Sand | | 0.471 | | Sand | | 0.273 | |
| | | | | | | | | | | | |
| Little Stour | Sand | Silt | | Little Stour | Sand | Silt | | Little Stour | Sand | Silt | |
| Gravel | 0.446 | <0.001 | | Gravel | 0.948 | 0.557 | | Gravel | 0.908 | 0.465 | |
| Sand | | <0.001 | | Sand | | 0.466 | | Sand | | 0.491 | |
| | | | | | | | | | | | |
| Mill Stream | Sand | Silt | | Mill Stream | Sand | Silt | | Mill Stream | Sand | Silt | |
| Gravel | <0.001 | 0.214 | | Gravel | <0.001 | <0.001 | | Gravel | <0.001 | <0.001 | |
| Sand | | <0.001 | | Sand | | <0.001 | | Sand | | 0.004 | |
| | | | | | | | | | | | |
| Wool Stream | Sand | Silt | | Wool Stream | Sand | Silt | | Wool Stream | Sand | Silt | |
| Gravel | <0.001 | 0.677 | | Gravel | 0.018 | <0.001 | | Gravel | 0.006 | <0.001 | |
| Sand | | <0.001 | | Sand | | 0.207 | | Sand | | 0.122 | |
| | | | | | | | | | | | |
| All data | Sand | Silt | | All data | Sand | Silt | | All data | Sand | Silt | |
| Gravel | <0.001 | 0.074 | | Gravel | <0.001 | <0.001 | | Gravel | <0.001 | <0.001 | |
| Sand | | 0.009 | | Sand | | 0.089 | | Sand | | 0.365 | |
| | | | | | | | | | | | |
| Functional Richness | | | | Functional Evenness | | | | Functional Divergence | | | |
| | | | | | | | | | | | |
| Frome | Sand | Silt | | Frome | Sand | Silt | | Frome | Sand | Silt | |
| Gravel | 0.331 | 0.116 | | Gravel | 0.035 | 0.189 | | Gravel | 0.807 | 0.357 | |
| Sand | | 0.017 | | Sand | | 0.376 | | Sand | | 0.551 | |
| | | | | | | | | | | | |
| Gadder | Sand | Silt | | Gadder | Sand | Silt | | Gadder | Sand | Silt | |
| Gravel | 0.248 | 0.290 | | Gravel | 0.086 | 0.359 | | Gravel | 0.450 | 0.466 | |
| Sand | | 0.956 | | Sand | | 0.451 | | Sand | | 0.999 | |
| | | | | | | | | | | | |
| Little Stour | Sand | Silt | | Little Stour | Sand | Silt | | Little Stour | Sand | Silt | |
| Gravel | 0.480 | 0.340 | | Gravel | 0.485 | <0.001 | | Gravel | 0.383 | 0.098 | |
| Sand | | 0.782 | | Sand | | <0.001 | | Sand | | 0.383 | |
| | | | | | | | | | | | |
| Mill Stream | Sand | Silt | | Mill Stream | Sand | Silt | | Mill Stream | Sand | Silt | |
| Gravel | <0.001 | 0.015 | | Gravel | 0.029 | 0.300 | | Gravel | 0.034 | 0.200 | |
| Sand | | <0.001 | | Sand | | 0.238 | | Sand | | <0.001 | |
| | | | | | | | | | | | |
| Wool Stream | Sand | Silt | | Wool Stream | Sand | Silt | | Wool Stream | Sand | Silt | |
| Gravel | 0.021 | 0.014 | | Gravel | 0.526 | 0.511 | | Gravel | 0.406 | <0.001 | |
| Sand | | 0.892 | | Sand | | 0.221 | | Sand | | <0.001 | |
| | | | | | | | | | | | |
| All data | Sand | Silt | | All data | Sand | Silt | | All data | Sand | Silt | |
| Gravel | <0.001 | 0.247 | | Gravel | 0.002 | 0.005 | | Gravel | 0.953 | <0.001 | |
| Sand | | <0.001 | | Sand | | 0.767 | | Sand | | <0.001 | |

## Slide 5
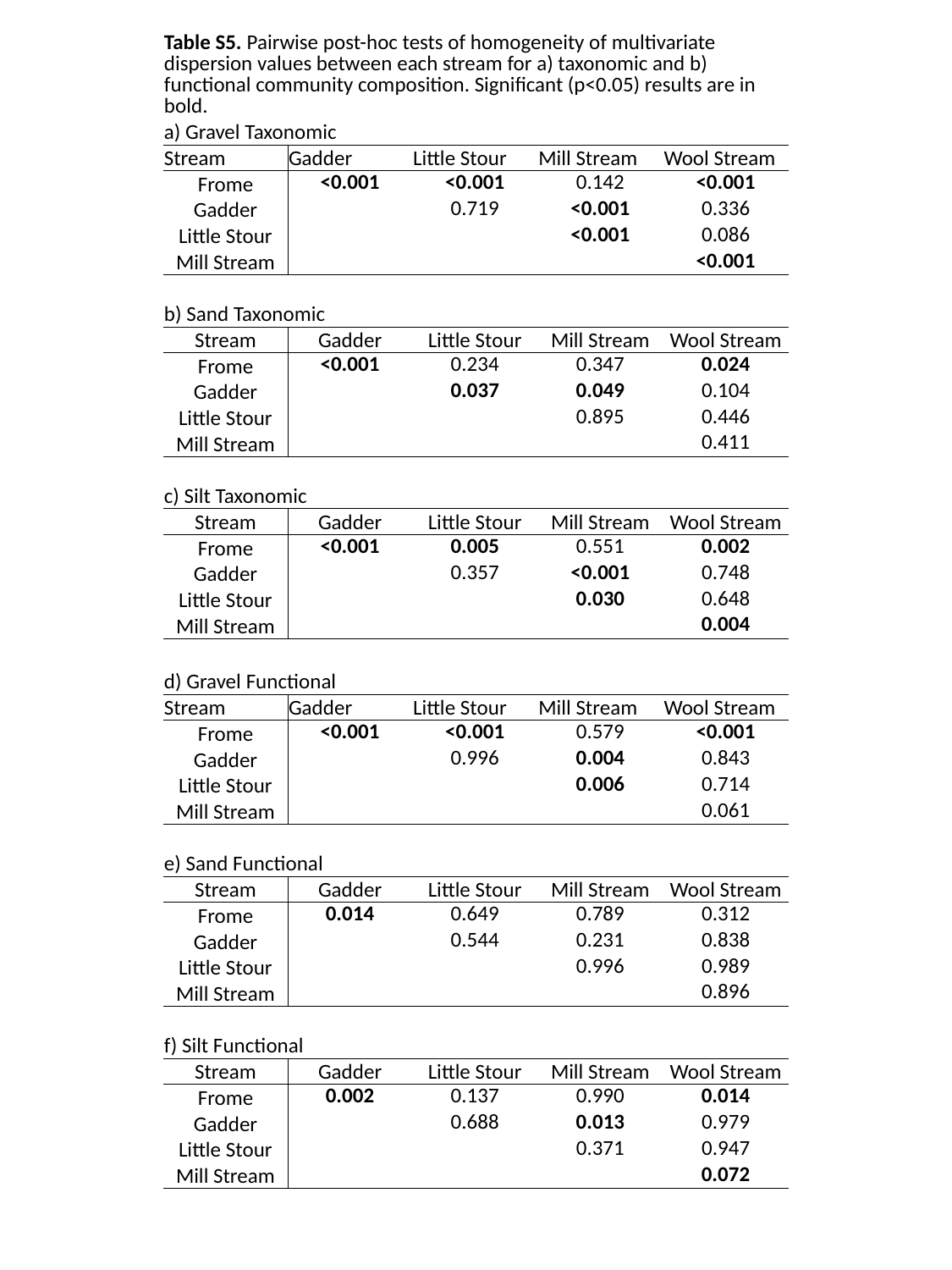

| Table S5. Pairwise post-hoc tests of homogeneity of multivariate dispersion values between each stream for a) taxonomic and b) functional community composition. Significant (p<0.05) results are in bold. | | | | |
| --- | --- | --- | --- | --- |
| a) Gravel Taxonomic | | | | |
| Stream | Gadder | Little Stour | Mill Stream | Wool Stream |
| Frome | <0.001 | <0.001 | 0.142 | <0.001 |
| Gadder | | 0.719 | <0.001 | 0.336 |
| Little Stour | | | <0.001 | 0.086 |
| Mill Stream | | | | <0.001 |
| | | | | |
| b) Sand Taxonomic | | | | |
| Stream | Gadder | Little Stour | Mill Stream | Wool Stream |
| Frome | <0.001 | 0.234 | 0.347 | 0.024 |
| Gadder | | 0.037 | 0.049 | 0.104 |
| Little Stour | | | 0.895 | 0.446 |
| Mill Stream | | | | 0.411 |
| | | | | |
| c) Silt Taxonomic | | | | |
| Stream | Gadder | Little Stour | Mill Stream | Wool Stream |
| Frome | <0.001 | 0.005 | 0.551 | 0.002 |
| Gadder | | 0.357 | <0.001 | 0.748 |
| Little Stour | | | 0.030 | 0.648 |
| Mill Stream | | | | 0.004 |
| | | | | |
| d) Gravel Functional | | | | |
| Stream | Gadder | Little Stour | Mill Stream | Wool Stream |
| Frome | <0.001 | <0.001 | 0.579 | <0.001 |
| Gadder | | 0.996 | 0.004 | 0.843 |
| Little Stour | | | 0.006 | 0.714 |
| Mill Stream | | | | 0.061 |
| | | | | |
| e) Sand Functional | | | | |
| Stream | Gadder | Little Stour | Mill Stream | Wool Stream |
| Frome | 0.014 | 0.649 | 0.789 | 0.312 |
| Gadder | | 0.544 | 0.231 | 0.838 |
| Little Stour | | | 0.996 | 0.989 |
| Mill Stream | | | | 0.896 |
| | | | | |
| f) Silt Functional | | | | |
| Stream | Gadder | Little Stour | Mill Stream | Wool Stream |
| Frome | 0.002 | 0.137 | 0.990 | 0.014 |
| Gadder | | 0.688 | 0.013 | 0.979 |
| Little Stour | | | 0.371 | 0.947 |
| Mill Stream | | | | 0.072 |
| | | | | |

## Slide 6
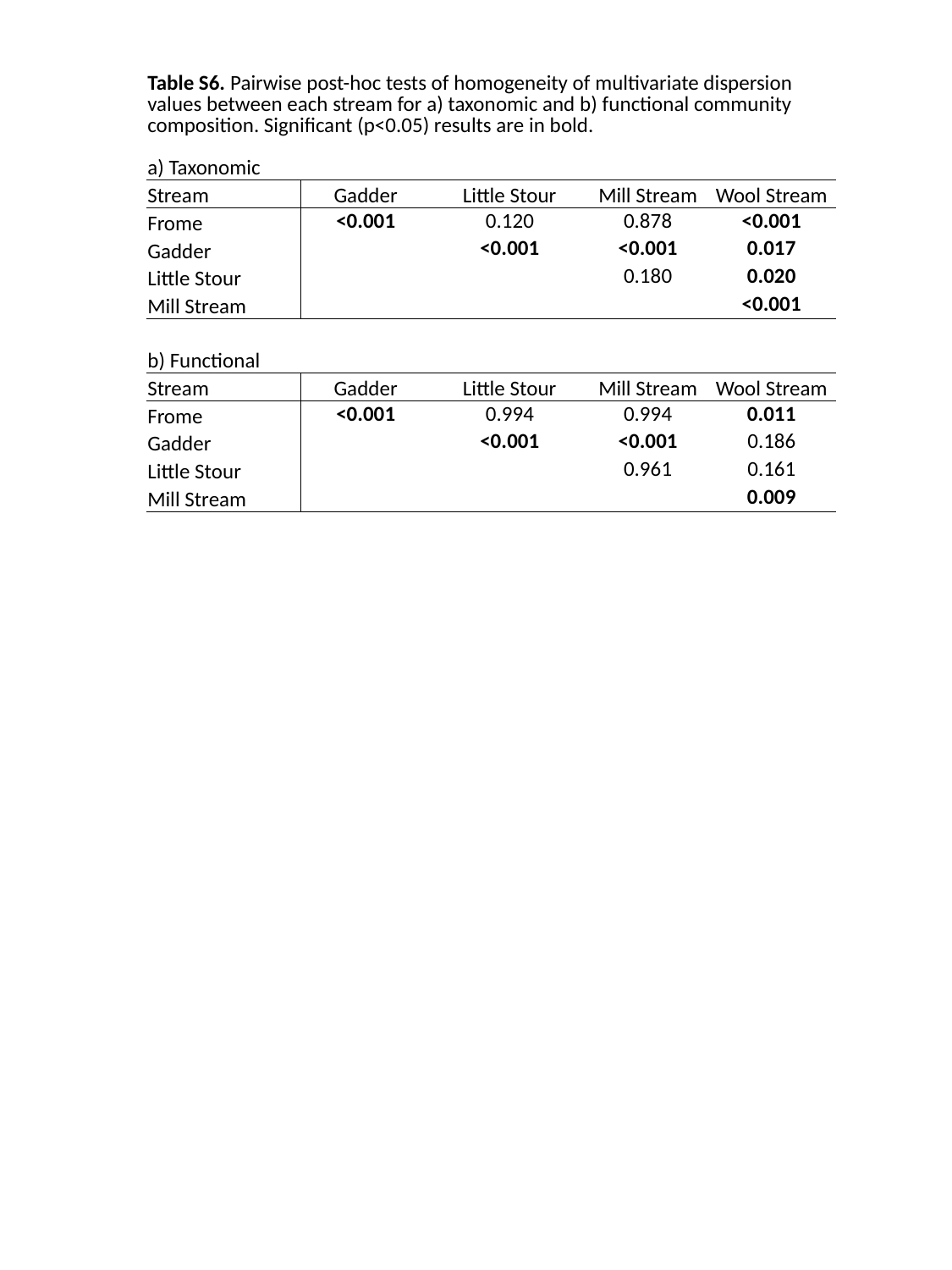

| Table S6. Pairwise post-hoc tests of homogeneity of multivariate dispersion values between each stream for a) taxonomic and b) functional community composition. Significant (p<0.05) results are in bold. a) Taxonomic | | | | |
| --- | --- | --- | --- | --- |
| Stream | Gadder | Little Stour | Mill Stream | Wool Stream |
| Frome | <0.001 | 0.120 | 0.878 | <0.001 |
| Gadder | | <0.001 | <0.001 | 0.017 |
| Little Stour | | | 0.180 | 0.020 |
| Mill Stream | | | | <0.001 |
| | | | | |
| b) Functional | | | | |
| Stream | Gadder | Little Stour | Mill Stream | Wool Stream |
| Frome | <0.001 | 0.994 | 0.994 | 0.011 |
| Gadder | | <0.001 | <0.001 | 0.186 |
| Little Stour | | | 0.961 | 0.161 |
| Mill Stream | | | | 0.009 |

## Slide 7
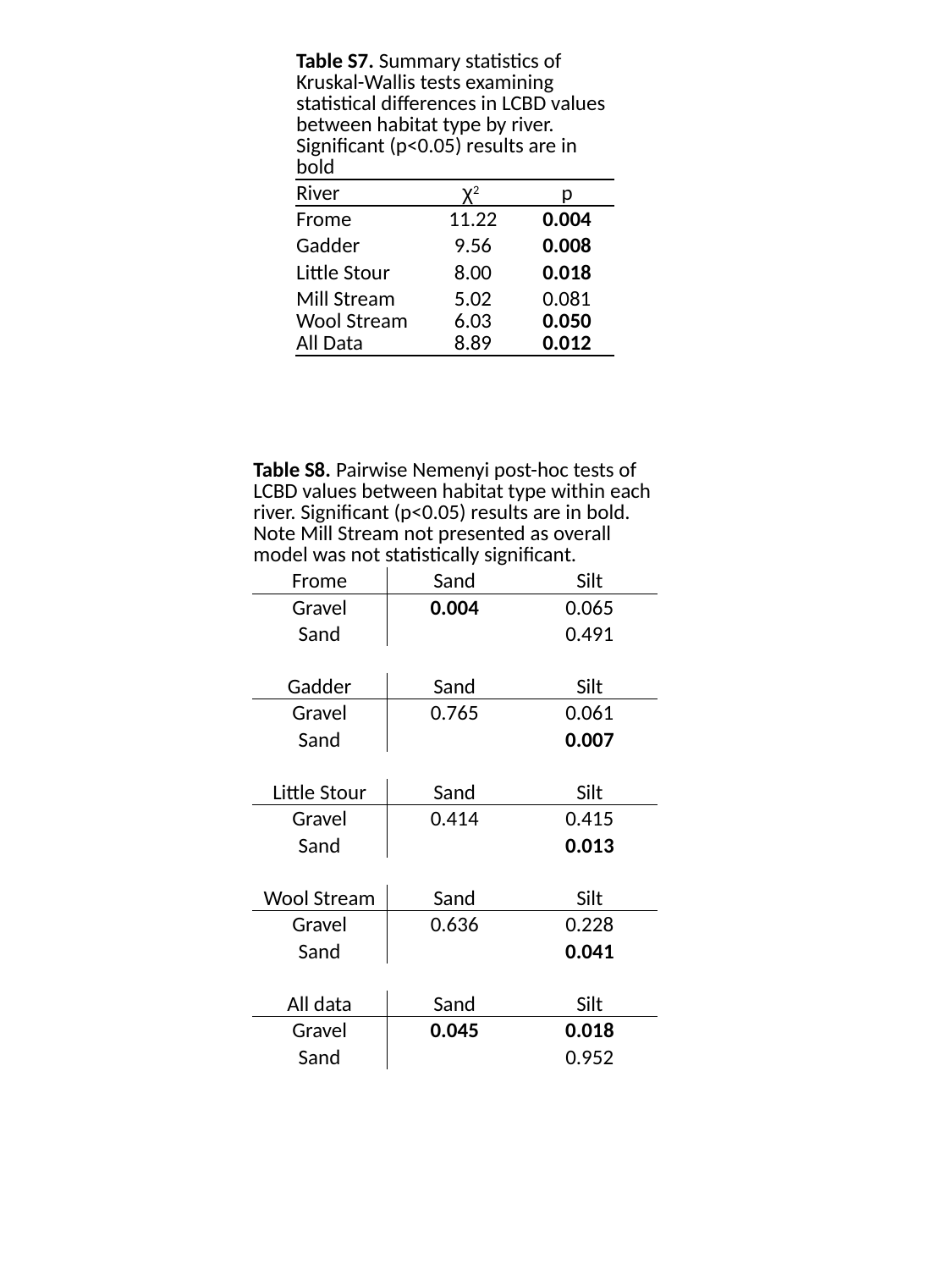

| Table S7. Summary statistics of Kruskal-Wallis tests examining statistical differences in LCBD values between habitat type by river. Significant (p<0.05) results are in bold | | |
| --- | --- | --- |
| River | χ2 | p |
| Frome | 11.22 | 0.004 |
| Gadder | 9.56 | 0.008 |
| Little Stour | 8.00 | 0.018 |
| Mill Stream | 5.02 | 0.081 |
| Wool Stream | 6.03 | 0.050 |
| All Data | 8.89 | 0.012 |
| Table S8. Pairwise Nemenyi post-hoc tests of LCBD values between habitat type within each river. Significant (p<0.05) results are in bold. Note Mill Stream not presented as overall model was not statistically significant. | | |
| --- | --- | --- |
| Frome | Sand | Silt |
| Gravel | 0.004 | 0.065 |
| Sand | | 0.491 |
| | | |
| Gadder | Sand | Silt |
| Gravel | 0.765 | 0.061 |
| Sand | | 0.007 |
| | | |
| Little Stour | Sand | Silt |
| Gravel | 0.414 | 0.415 |
| Sand | | 0.013 |
| | | |
| Wool Stream | Sand | Silt |
| Gravel | 0.636 | 0.228 |
| Sand | | 0.041 |
| | | |
| All data | Sand | Silt |
| Gravel | 0.045 | 0.018 |
| Sand | | 0.952 |

## Slide 8
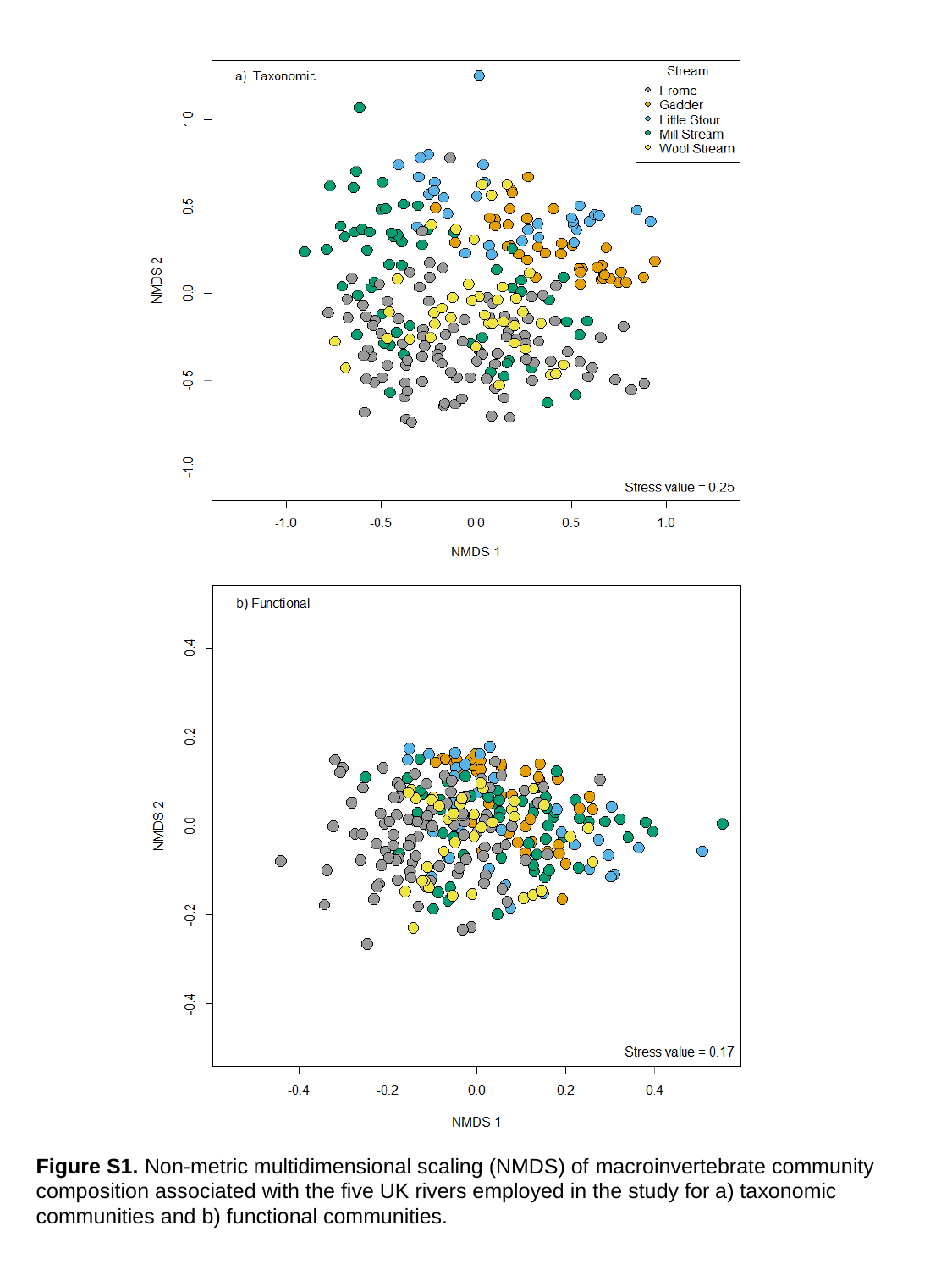

Figure S1. Non-metric multidimensional scaling (NMDS) of macroinvertebrate community composition associated with the five UK rivers employed in the study for a) taxonomic communities and b) functional communities.

## Slide 9
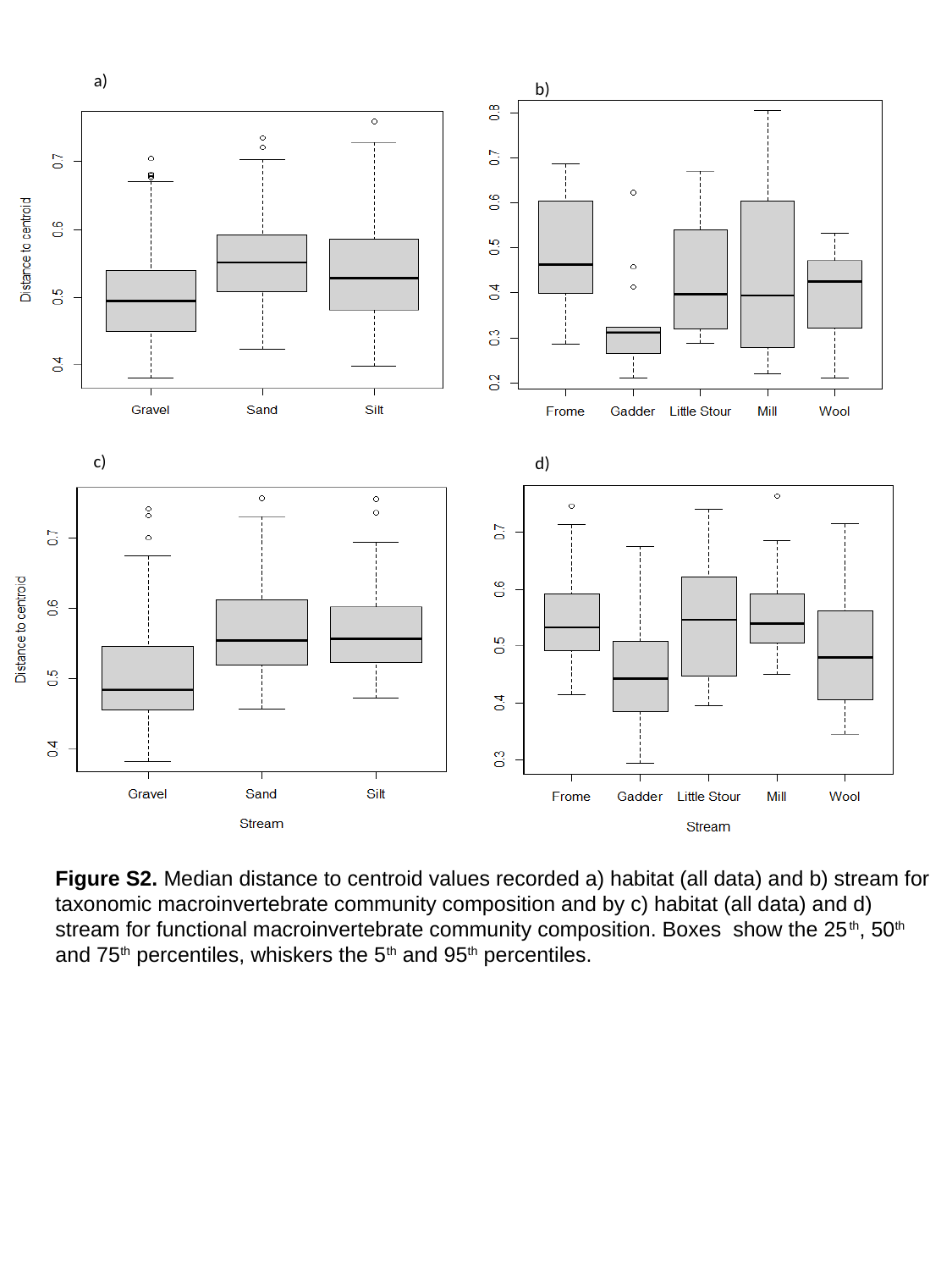

a)
b)
c)
d)
Figure S2. Median distance to centroid values recorded a) habitat (all data) and b) stream for taxonomic macroinvertebrate community composition and by c) habitat (all data) and d) stream for functional macroinvertebrate community composition. Boxes  show the 25th, 50th and 75th percentiles, whiskers the 5th and 95th percentiles.

## Slide 10
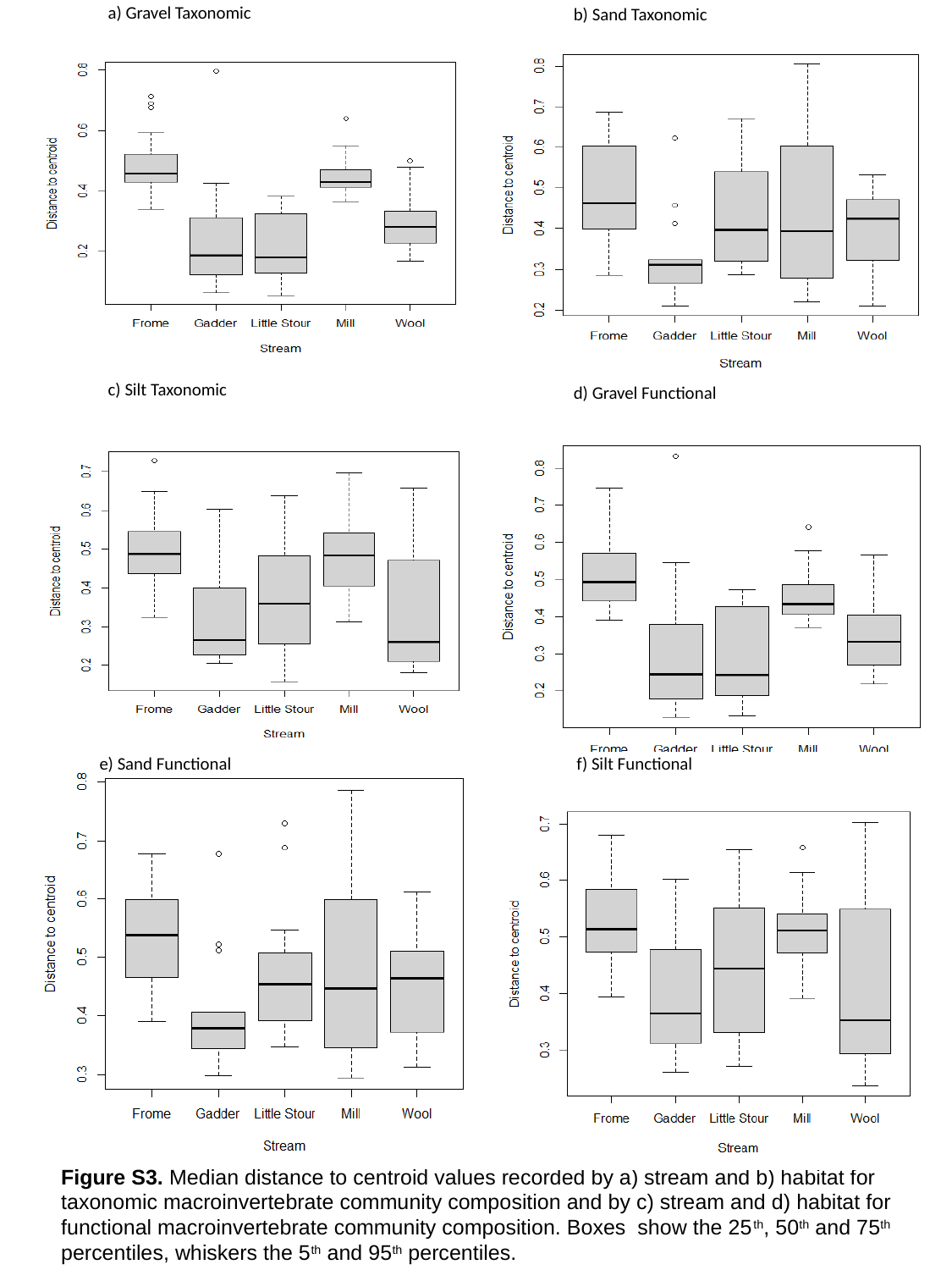

a) Gravel Taxonomic
b) Sand Taxonomic
c) Silt Taxonomic
d) Gravel Functional
e) Sand Functional
f) Silt Functional
Figure S3. Median distance to centroid values recorded by a) stream and b) habitat for taxonomic macroinvertebrate community composition and by c) stream and d) habitat for functional macroinvertebrate community composition. Boxes  show the 25th, 50th and 75th percentiles, whiskers the 5th and 95th percentiles.
